# Food safety knowledge, attitudes and practices of restaurant food handlers in a lower‐middle‐income country

**DOI:** 10.1002/fsn3.1454

**Published:** 2020-02-13

**Authors:** France Ncube, Artwell Kanda, Morleen Chijokwe, Goden Mabaya, Tendayi Nyamugure

**Affiliations:** ^1^ Department of Environmental Science Bindura University of Science Education Bindura Zimbabwe; ^2^ Department of Agricultural Economics, Education and Extension Bindura University of Science Education Bindura Zimbabwe

**Keywords:** Attitudes, food handler, food safety knowledge, practices, restaurant

## Abstract

Global research attention appears to be focused predominantly on self‐reported than observed food safety practices. The purpose of this study was to determine the food safety knowledge, attitudes, and self‐reported and observed practices of food handlers in 22 urban restaurants in Zimbabwe. A piloted questionnaire was used to gather qualitative data regarding socio‐demographic variables, food safety knowledge (FSK), attitudes, and self‐reported food handling practices (SRFHPs). A predesigned checklist was used to observe the food handling practices. FSK scores were significantly higher in food handlers who received basic food safety training compared to those who did not (*p* < .05). No differences in food safety knowledge and attitudes were noted based on the socio‐demographic characteristics of the food handlers (*p* > .05). A significant positive correlation was observed between FSK and attitudes (*r*_s_ = 0.371, *p* < .05), FSK and SRFHPs (*r*_s_ = 0.242, *p* < 0.05), FSK and observed food handling practices (OFHPs) (*r*_s_ = 0.254, *p* < .05), attitudes and SRFPs (*r*_s_ = 0.229, *p* < .05), and attitudes and OFHPs (*r*_s_ = 0.263, *p* < .05). About half of the food handlers washed their hands in sinks meant for washing cutlery, 57% did not use approved hand drying methods, and 19.8% did not adequately thaw frozen foods. Food was commonly defrosted either under room temperature or using hot water (>45°C). Results suggest a need for mandatory basic and advanced training to improve the food safety knowledge, attitudes, and practices.

## Graphical abstract

This article determined the food safety knowledge, attitudes, and practices of restaurant handlers in 22 urban restaurants in Zimbabwe. A significant positive correlation was observed between (i) food safety knowledge and attitudes, (ii) attitudes and self‐reported food safety practices, and (iii) attitudes and observed food safety practices.

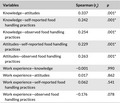

## 1 INTRODUCTION

About 2.2 million people die annually from food‐ and water‐borne diarrheal diseases (WHO, 2013). The prevalence rate of food‐borne diseases is higher in low‐income than in high‐income countries (WHO, 2015). The higher prevalence in low‐income countries has been attributed to the use of unsafe water for cleaning and food processing, substandard food production processes and poor food handling, lack of adequate food storage facilities, and inadequate or poorly enforced food safety laws (WHO, 2015). Food‐borne diseases constitute a substantial strain on health‐care systems, trade and tourism (WHO, 2013). They reduce economic productivity and threaten livelihoods (WHO, 2013). International food safety interventions such as the Hazard Analysis Critical Control Point (HACCP), the Codex Alimentarius (2003), ISO 22000:2018, and the WHO Food Safety Strategic plan (2013–2022) emphasize the need to identify and rectify food safety inadequacies. In Zimbabwe, there is currently no legal requirement for food premises to be HACCP or ISO 22000 certified. Resultantly, these preventive food safety systems are currently not widely applied in the food service sector.

Studies predominantly associate food poisoning with poor food handling practices (Clayton, 2002; McIntyre, Vallaster, Wilcott, Henderson, & Kosatsky, 2013). Food handlers seem to be a major source and means of food contamination, particularly in ready to eat food, such as that served in restaurants. Soares, Almeida, Cerqueira, Carvalho, and Nunes (2012) highlighted that the majority of food handlers had hand contamination with coagulase‐positive staphylococci. In addition, an investigation by Lee, Halim, Thong, and Chai (2017) showed that 48% of food handlers whose hands were swabbed for microbiological assessment had salmonella whereas about two‐thirds had ≥ 20cfu of total aerobic counts. Similarly, Illes, Toth, Dunay, Lehota, and Bittsanszky (2018) swabbed school kitchen utensils that come into contact with food and reported that most utensils were contaminated with mesophilic aerobic bacteria. They reported a strong correlation between food handlers’ knowledge on food hygiene and microbiological contamination of the utensils. There is no evidence that the prevalence rate of food‐borne diseases is diminishing (Soares et al., 2012). This underlines the need for further studies concerning the determinants of safe food handling behavior.

Knowledge, attitudes, and self‐reported practices (KAP) of food handlers on food safety has received much global research (Rebouças et al., 2016; Al‐Shabib, Mosilhey, & Husain, 2016; Bou‐Mitri, Mahmoud, Gerges, & Jaoude, 2018; Zanin, Cunha, Rosso, Capriles, & Stedefeldt, 2017). However, there are limited studies that used observation to investigate the food handling practices (da Cunha, Stedefeldt, & de Rosso, 2014; Soares et al., 2012; de Souza, de Azevedo, & Seabra, 2018). Self‐reported practices may not necessarily be the actual practiced food handling behavior (Bou‐Mitri et al., 2018; McIntyre et al., 2013), as they may include respondent bias in the study findings (Ncube, Ncube, & Voyi, 2017).

Zimbabwe is a lower‐middle‐income country (World Bank, 2019). Its gross domestic product (GDP) per capita is 1.411 (World Bank, 2019). Restaurant food handlers in Zimbabwe are an understudied group with regards to food safety. There are no published local studies on this category of workers. Understanding the knowledge, attitudes, and practices of food safety by restaurant food handlers enables regulatory authorities to take evidence derived measures toward the provision of safe and wholesome food to the consumer. Such measures may include appropriate educational interventions that effectively address the food handlers’ knowledge gaps, attitudes, and practices on food safety (Gillespie, Little, & Mitchell, 2000; Illes et al., 2018). The current study was, therefore, conducted to assess the knowledge, attitudes, and practices (observed and self‐reported) of restaurants’ food handlers on food safety and the factors that influence the use of safe food handling practices.

## 2 MATERIALS AND METHODS

**Figure 1:**
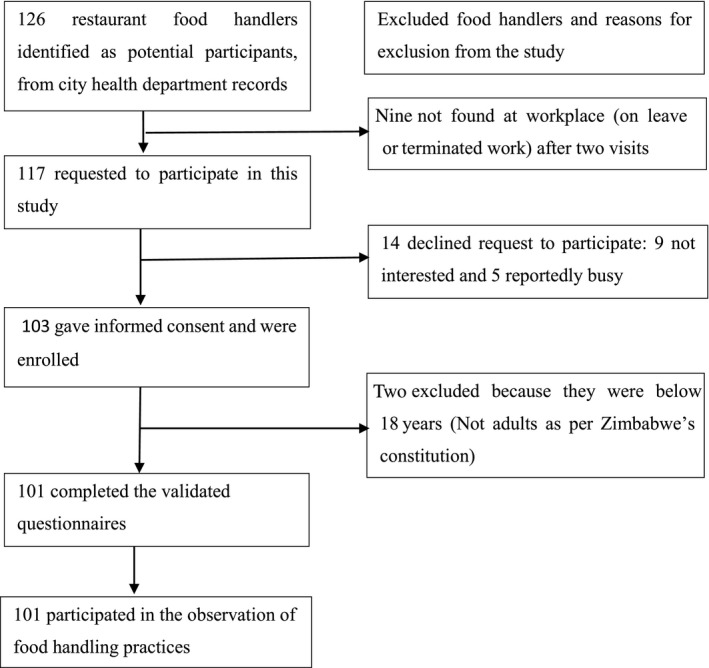
Sample selection process

A cross‐sectional study was carried out in Bindura town (17.30138 S, 31.31988 E) between January and May 2018. Bindura is the provincial town of Mashonaland Central Province, Zimbabwe. A total of 101 food handlers engaged in the preparation, serving, and sale of food were purposively selected from 22 commercial restaurants. About 77% of the restaurants were small‐sized and served up to 100 meals a day. The remainder were medium‐sized and served over 100 meals a day. Despite preparing, serving, and handling high‐risk foods (e.g., salads, eggs, meat, poultry, fish, and rice), none of the restaurants had a HACCP and/or ISO 22000 certification. The clientele comprised mainly lecturers and students from the three local universities, workers from various government departments and nongovernmental organizations, and workers from commercial and industrial sectors. Figure 1 illustrates the sample selection process. Data were collected using a questionnaire and an observation checklist guide.

### 2.1 Questionnaire

A structured questionnaire was developed based on the HACCP principles, elements assessed in similar past studies (Sharif, and AI‐Malki, 2010; McIntyre et al., 2013; Rebouças et al., 2016), and the requirements of Zimbabwe's food safety legislation (Ministry of Health and Child Care 1996a, 1996b). The questionnaire was designed to assess the knowledge, attitudes, and self‐reported work practices of food handlers on food safety. It was designed in English, translated to, and administered in the local language (*ChiShona*) and then retranslated to English for data analyses and reporting. Questionnaire translation was carried out independently by two native *ChiShona* speakers. They exchanged the translated versions and harmonized them through discussion (discussion meeting attended by two authors) to produce one improved document.

Kappa coefficients (k) were calculated to measure the level of interrater reliability. The raters were two professional food inspectors and were given the KAP questionnaires that comprised 55 items (20 items on food safety knowledge assessment, 20 on self‐reported practices and 15 items on food safety attitudes). They rated whether each item in the questionnaire needed further revision to improve on clarity. The raters either said yes (where further revision was needed) or no (where no further revision was need). Then, kappa coefficients were calculated using a procedure described in literature (Cohen, 1960; Kottner & Dassen, 2008) and values ranged from 0.76 to 0.94, which demonstrated a good measure of reliability.

Participants were advised not to write their names or any form personal identification details in order to ensure anonymity and to reduce respondent bias. Confidentiality was guaranteed by informing participants that their individual responses were not to be accessible or communicated to management. To improve on the validity of the questionnaire, it was peer‐reviewed by two professional food inspectors (Abdul‐Mutalib et al., 2012; Soares et al., 2012) and piloted (Pichler, Ziegler, Aldrian, & Allerberger, 2014; Soares et al., 2012; Woh, Thong, Behnke, Lewis, & Jain, 2016) with 25 respondents (24.8% of the sample size). The revised version of the questionnaire was self‐administered to the study participants. Prior to administration of the questionnaire, a 10–15 min training of the respondents was carried out on how to answer the questionnaire, the importance of the reliability and completeness of collected information, the study's objectives and the respondents rights.

Questionnaires were completed in the presence of the trained research assistants in order to preclude the respondents from being assisted by workmates and looking up information (Pichler et al., 2014). In circumstances where a respondent was unable to read or write, a trained research assistant interviewed the respondent and completed the questionnaire. Each questionnaire had five sections: demographic information (age, gender, educational level, and work responsibility), work history (period employed as a restaurant food worker, hours worked: per day and week), knowledge, attitudes, and self‐reported work practices. The sections that assessed knowledge (20 items) and self‐reported practices (20 items) consisted of questions pertaining to food spoilage, storage, and the sources and means of food contamination. The attitude assessment section of the questionnaire comprised 15 items. The questions pertained to the food handlers’ perceptions on food safety training, food sanitation, and supervision, responsibilities toward preventing food contamination, and treatment seeking behavior for food transmitted diseases. It took about 10–15 min to complete each questionnaire. The KAP of participants were scored using a procedure described in past studies (Al‐Shabib, Mosilhey, & Husain, 2016; Sharif & Al‐Malki, 2010). A four (4) was accorded for a correct response or practice while a zero (0) was given for an incorrect one. A measurement scale (0 – 4) was applied to interpret the mean KAP scores: 0 = poor, 1 = unsatisfactory, 2 = average, 3 = satisfactory, and 4 = excellent.

### 2.2 Observation checklist guide

Visual observations of the food handling practices were carried out by three trained research assistants under the supervision of two certified food inspectors. A predesigned observation checklist guide was used. The guide contained same questions that assessed self‐reported food handling practices in the questionnaire described earlier on. It provided some form of methodological triangulation by validating information pertaining to the self‐reported practices.

### 2.3 Statistical analyses

Analyses were performed using the Statistical Programme for Social Sciences (SPSS) version 20.0. A chi‐squared test was performed to determine whether the food handlers’ food safety knowledge and attitudes differed with education level, age, gender, and work experience and to determine whether there were significant differences between self‐reported and observed food handling practices. The scores with respect to food handlers’ food safety knowledge and attitudes were summarized using descriptive statistics (mean and standard deviation). The Spearman's correlation test was carried out to determine the association among knowledge, attitudes, self‐reported practices, observed practices, and work experience. Statistically significant differences were considered at 95% level of confidence (*p* < .05).

## 3 RESULTS AND DISCUSSION

### 3.1 Association of socio‐demographic characteristics with food safety knowledge and attitudes

Table 1 shows the association of socio‐demographic characteristics with food safety knowledge and attitudes. No differences in food safety knowledge and attitudes were noted based on the gender, age, educational level, and work experience of the food handlers (*p* > .05). This is consistent with a study by Abdul‐Mutalib et al. (2012), which did not find a significant association between the respondents’ knowledge level and socio‐demographic characteristics. On the contrary, McIntyre et al. (2013) reported that socio‐demographic factors such as the length of work experience in the food industry and food handlers’ level of education were significantly associated with improved food safety knowledge. More studies are required to better understand the influence of socio‐demographic factors on the food safety knowledge and attitudes of food handlers. Most of the participants (81.2%) in the current study were females aged between 18 and 37 years (27.34 ± 6.8 years) and were married. In the Zimbabwean context, the preponderance of women labor in restaurants is not surprising as this category of the population is traditionally considered primarily responsible for household food preparation activities. Consistent with this tradition, the food service sector is generally dominated by the female labor force. The large number of female handlers in our study is consistent with reports from similar past studies (da Cunha et al., 2014; Lee et al., 2017; Martins, Hogg, & Otero, 2012). Over 50% of the women had up to 3 years of work experience as a food handler in the same restaurant. The lack of varied food handling experience may increase the risk of food contamination due to nonuse of good manufacturing practices. About 32% of the food handlers had not gone beyond primary education, with over 80% (26) of them being female. Given that women perform various food handling activities at work and at home, it appears very critical to take into account their educational status and literacy level when designing and implementing food safety training programmes.

**Table 1:** Association of socio‐demographic variables with food safety knowledge and attitudes

Variable	*n* (%)	Food safety knowledge		χ^2^	*p*	Food safety attitudes		χ^2^	*p*
		Satisfactory *n* (%)	Inadequate *n* (%)			Negative *n* (%)	Positive *n* (%)		
Gender									
Male	19 (18.8)	6 (31.6)	13 (68.4)	0.174	.677	1 (5.3)	18 (94.7)	0.019	.890
						5 (6.1)	77 (93.9)		
Female	82 (81.2)	22 (26.8)	60 (73.2)						
Age (Mean±SD: 27.85±7.2 years)									
≤25	46 (45.5)	11 (23.9)	35 (76.1)	0.612	.434	3 (6.5)	43 (93.5)	0.051	.821
> 25	55 (54.5)	17 (30.9)	38 (69.1)			3 (5.5)	52 (94.5)		
Educational level (Mean±SD: 9.59±2.3 years of education)									
Primary and below	32 (31.7)	8 (25.0)	24 (75.0)	0.173	.677	0 (0)	32 (100)	2.958	.085
						6 (8.7)	63 (91.3)		
Secondary and above	69 (68.3)	20 (29.0)	49 (71.0)						
Work experience as a food handler (Mean±SD: 3.09±2.6 years)									
≤2 (Inexperienced)	47 (46.5)	13 (27.7)	34 (72.3)	0.001	.989	3 (6.4)	44 (93.6)	0.031	.861
						3 (5.6)	51 (94.4)		
>2 (Experienced)	54 (53.5)	15 (27.8)	39 (72.2)						
Basic training on food safety									
Trained	36 (35.6)	2 (5.6)	34 (94.4)	13.718	.001*	0 (0)	36 (100)	3.533	.060
						6 (5.9)	59 (94.1)		
Not trained	65 (64.4)	26 (40.0)	39 (60.0)						
HACCP training									
Trained	4 (4)	0 (0)	4 (100)	1.598	.206	0 (0)	4 (100)	0.263	1.000
						6 (6.2)	91 (93.8)		
Not trained	97 (96)	28 (28.9)	73 (72.3)						

* *p* < .05, satisfactory means >50% of questions in Table 2 correctly answered, positive means >50% of questions in Table 3 correctly answered.

The food safety knowledge of food handlers who had received basic food training significantly differed from those who did not (*p* < .05; Table 1). Similarly, McIntyre et al. (2013) reported significantly higher knowledge scores for trained compared with untrained food handlers. This suggests that to strengthen the food safety knowledge of food handlers, restaurant managers need to provide basic relevant training. The majority of food handlers neither received basic food safety training (64.4%) nor HACCP training (96%), as shown in Table 1. This suggests that the food handlers’ food safety knowledge reported in the present study could have been obtained from other sources such as workmates, media, and formal education. Unlike in British Colombia, Brazil, Malaysia, European countries, and the United States (McIntyre et al., 2013; da Cunha et al., 2014; Lee et al., 2017; EU Regulation 2004; Food & Drug Administration, 2017) food safety training is not yet mandatory in Zimbabwe (Ministry of Health and Child Care, 1996a,b), which may account for the low number of trained food handlers. We recommend widening the scope of Zimbabwe's food safety laws to include a requirement for managers in the food service sector to provide periodic food safety training to food handlers. Such training should be provided every 6–12 months (da Cunha, Rosso, Pereira, & Stedefeldt, 2019), and its effectiveness should be evaluated (Soares et al., 2012; ISO, 2018). Further, the food safety training should place more emphasis on use of techniques that promote behavioral change, and acquisition practical skills for the performance of recommended food hygiene procedures (da Cunha et al., 2019; Egan et al., 2007; Medeiros, Cavalli, Salay, & Proença, 2011; Reynolds & Dolasinski, 2019). In addition, managers of food premises should provide motivation and support to food handlers to ensure the success of food safety training (Al‐Shabib et al., 2016).

### 3.2 Knowledge of participants on food safety

The descriptive statistics concerning the study participants’ food safety knowledge is shown in Table 2. The highest knowledge score was 3.96 ± 0.40 and pertained to the risk to food contamination by food handlers suffering from diseases such as diarrhea, sore throat, syphilis, and flu. This shows that participants understood the risk to food contamination that an unhealthy food handler posed. Similar studies have demonstrated that food handlers have good knowledge with regards to this issue (da Cunha et al., 2014; McIntyre et al., 2013; Pichler et al., 2014). However, some studies reported that food handlers lacked adequate knowledge about the risk of contamination of food by diarrheal food handlers (Clayton, 2002; Osaili, Obeidat, Jamous, & Bawadi, 2011).

**Table 2:** Food handlers’ knowledge on food safety

Statement	Agree *n* (%)	Score (Mean ± *SD*)
The safe operating temperature for a refrigerator is 1–5°C	73 (72.3)	2.89 ± 1.80
Refrigeration and freezing do not destroy most bacteria	60 (39.6)	1.97 ± 1.58
Reheating rice contributes to bacterial food poisoning (BFP)	40 (57.4)	2.30 ± 1.99
Refreezing defrosted food contributes to BFP	61 (60.4)	2.42 ± 1.97
Inadequate thawing of food can contribute to BFP	87 (86.1)	3.45 ± 1.39
Eating undercooked food such as meat may contribute to BFP	83 (82.2)	3.29 ± 1.54
Use of separate cutlery to prepare or handle raw and cooked foods minimizes food contamination	82 (81.2)	3.25 ± 1.67
Contact between raw and cooked foods contributes to food contamination	86 (85.1)	3.41 ± 1.43
Not wearing rings, watches, necklaces minimizes food contamination	90 (89.1)	3.56 ± 1.25
The temperature range 5–47°C is suitable for the growth of most bacteria that spoil food	73 (73.3)	2.93 ± 1.78
Pets in food premises can contaminate food	86 (85.1)	3.41 ± 1.43
Cleaning and sanitizing utensils reduces the risk of food contamination	90 (89.1)	3.56 ± 1.25
Wooden chopping boards are a high risk for food contamination	72 (71.3)	2.85 ± 1.82
Using gloves to handle raw foods reduces the risk of food contamination	69 (68.3)	2.73 ± 1.87
Eating, drinking, talking, and smoking when preparing or serving food increase the risk of food contamination.	79 (78.2)	3.13 ± 1.66
Use of dish towels to wipe hands can contaminate food	58 (57.4)	2.30 ± 1.99
Handwashing in sinks for washing cutlery increases the risk of food contamination	69 (68.3)	2.73 ± 1.87
Keeping nails short and unpainted reduces the risk of food contamination	94 (93.1)	3.72 ± 1.02
A healthy food handler can be a carrier of infectious food‐borne diseases	79 (78.2)	3.13 ± 1.66
A food handler suffering from diseases such as diarrhea, sore throat, syphilis and flu poses a risk of food contamination	100 (99)	3.96 ± 0.40
Average	76.6 (75.8)	3.03 ± 1.71

Note

Scores (0‐4): 0 = least score (poor); 1 = unsatisfactory, 2 = average, 3 = satisfactory, 4 = highest score (excellent).

Abbreviation: *SD*, standard deviation.

At least 85% of the participants had satisfactory to excellent (3–4) mean knowledge scores concerning the statements: inadequate thawing of food can contribute to bacterial food poisoning (BFP), contact between raw and cooked foods contributes to food contamination, not wearing rings, watches, necklaces minimizes food contamination, cleaning, and sanitizing utensils reduces the risk of food contamination and keeping nails short and unpainted reduces the risk of food contamination (Table 2). McIntyre et al. (2013) reported that most food handlers had excellent knowledge about how to thaw frozen foods such as red meats. Smigic et al. (2016) and Bou‐Mitri et al. (2018) demonstrated that over 90% of the food handlers knew that separate storage of cooked and raw foods is necessary to prevent bacterial food contamination. In Brazil, da Cunha et al., (2014) reported that about 92% of the food handlers knew that use of earrings, rings, and watches could contribute to food contamination. In Portugal, Martins et al. (2012) reported significantly higher food handlers’ knowledge on surface and utensils hygiene than the overall food hygiene knowledge.

The least mean food safety knowledge score of 2.30 ± 1.99 was recorded in response to the use of dish towels to wipe hands as source of food contamination (Table 2). This means that the food handlers lacked knowledge regarding cross contamination of dish towels by pathogens from fingers, nails, and palms from the drying with dish towels. About 32% of the food handlers were not aware that handwashing in sinks for washing cutlery increases the risk of food contamination. This highlights the need to strengthen food handlers’ food safety knowledge through the provision of basic food safety training, as the majority of them never attended such training (Table 1). However, such training should be carefully designed and implemented in order to yield significant improvements in food handlers’ attitudes and/or practices. Zanin et al. (2017) conducted review of food handlers' knowledge, attitudes, and practices and reported that in about 50% of the reviewed studies (*n* = 36) knowledge and/or attitudes were commonly not translated into practices. Consequently, to enhance transformation of food safety knowledge and/or attitudes into practices, factors such as the training strategy and characteristics of training venue should be taken into account when designing and implementation food safety training programmes (Zanin et al., 2017).

At least 30% of the food handlers in the current study lacked awareness that the use gloves to handle raw foods reduces the risk of food contamination, as shown in Table 2. Bou‐Mitri et al. (2018) also reported substantial deficits in food safety knowledge of hospital food handlers, with regards to this particular issue. Approximately 40% of the food handlers did not know that refreezing defrosted food contributes to bacterial food poisoning (Table 2). This is consistent with previous food safety studies (Abdul‐Mutalib et al., 2012; Sani & Siow, 2014).

About 73% of the food handlers indicated that the safe operating temperature for a refrigerator is 1–5°C, as shown in Table 2. Previous studies (Martins et al., 2012; McIntyre et al., 2013; Pichler et al., 2014) showed that food handlers lacked adequate knowledge concerning the recommended operation temperatures of food refrigerators. This reflects a training need to improve on the awareness about the proper cold storage of perishable food. Past studies have demonstrated that training programmes improve food handlers’ knowledge on food safety hazards (da Cunha et al., 2014; McIntyre et al., 2013; Pichler et al., 2014). Da Cunha et al. (2014) recommend that the food safety training should be conducted more regularly, such as every six to twelve months, in order to refresh food handlers on learnt content. In recognition of the value of food safety training the European Union requires that member states offer the training at least annually to food handlers (Regulation EC, 2004). However, in resource‐constrained settings, a more regular frequency, such as every quarter, may be required due to higher turn overs of workers. Restaurant food handlers may also benefit from advanced food safety training such as HACCP and ISO 22000:2018. Overall, the grand mean score concerning knowledge on food safety was 3.03 ± 1.71 and the average percentage of correct responses was 75.8% (Table 2). This is comparable with the work of da Cunha et al. (2014). Both studies cement the conclusion that food handlers’ food safety knowledge is insufficient and require major improvements as highlighted earlier on in this article.

### 3.3 Food safety attitudes

The food safety attitudes of restaurant food handlers are presented in Table 3. The present study indicates that over 80% of the food handlers had positive attitudes toward food safety training, observance of proper cleaning procedures and cutlery color codes, preventing food contamination, and task performance supervision. This is in agreement with earlier studies (McIntyre et al., 2013; da Cunha et al., 2014; Al‐Shabib, Mosilhey, & Husain., 2016). About 25% reported that they will not inform their supervisors if they suffer from diarrhea, wounds or cuts. In addition, 25.7% of them would not take sick leave if they have diarrhea, wounds or cuts. Such attitudes of food handlers may result in the spread of food‐borne diseases during food preparation, handling and serving. Noteworthy, food handlers have been reported to perceive their risk of spreading food‐borne diseases to be low, a phenomena termed optimistic bias (da Cunha et al., 2019). Resultantly, they may not consider themselves to be in need of food safety training and education (Jenner et al., 2006). We propose that restaurant managers should commit themselves to building a positive food safety culture (PFSC) among food handlers. A PFSC is defined as an organization's culture that is strongly supportive of food safety and is perceived to be important to the accomplishment of the organization's vision (Griffith, 2013). Improving the PFSC within an organization yields hygiene compliance not only of existing workers but also potentially for new ones (Griffith, 2013). Further studies are required to investigate the key factors that perpetuate the negative attitude toward issues such as reporting of illness and taking of sick leave, and to determine required remedies.

**Table 3:** Food handlers’ food safety attitudes

Statement	Agree *n* (%)	Score (Mean ± *SD*)
I am willing to learn about the basics of food hygiene and safety	95 (94.1)	3.76 ± 0.95
I think restaurant managers should organize advanced training such as hazard analysis critical control point for food handlers	84 (83.2)	3.33 ± 1.50
I will inform my supervisor if l have diarrhea, wounds or cuts	76 (75.2)	3.01 ± 1.74
I will take sick leave if l have diarrhea, wounds or cuts	75 (74.3)	2.97 ± 1.76
I try do my level best to always observe proper cleaning procedures	94 (93.1)	3.72 ± 1.02
I am willing to observe cutlery color codes for different uses e.g. red for red meat, green for vegetables.	91 (90.1)	3.60 ± 1.20
Preventing food contamination and spoilage is my key responsibility	95 (94.1)	3.76 ± 0.95
Expired food should never be consumed	86 (85.1)	3.41 ± 1.43
I consistently use gloves to handle nonpacked food even if my supervisor is absent	87 (86.1)	3.45 ± 1.34
I do not need incentives to do my best to prevent food contamination	94 (93.1)	3.72 ± 1.02
My number one reason for observing set food hygiene standards is not the fear of management’s disciplinary measures	90 (89.1)	3.56 ± 1.25
Swabbing of food handlers’ palms and nails is useful for assessing the effectiveness of handwashing	78 (77.2)	3.09 ± 1.69
Physical assessment of food handlers’ personal hygiene is important for minimizing food contamination	80 (79.2)	3.17 ± 1.63
Task performance supervision motivates me	84 (83.2)	3.33 ± 1.50
To be certain that food is safe to eat, one should cook it for the duration of the recommended time than smell or taste it	82 (81.2)	3.25 ± 1.57
Average	86 (85.2)	3.41 ± 1.42

Note

Scores (0–4): 0 = least score (poor); 1 = unsatisfactory, 2 = average, 3 = satisfactory, 4 = highest score (excellent).

Abbreviation: *SD*, standard deviation.

About 23% of the participants considered microbiological swabbing (surface sampling) of their palms and nails as not useful with regards to assessing the effectiveness of handwashing. Previous researches support the conclusion that food handlers’ palms and nails pose a major risk to food contamination. For example, in a study of food handlers working in school kitchens in Brazil, Soares et al. (2012) reported hand contamination with coagulase‐positive staphylococci. In addition, an investigation by Lee et al. (2017) showed that 48% of food handlers whose hands were swabbed for microbiological assessment had salmonella whereas about two‐thirds had ≥ 20cfu of total aerobic counts. Therefore, it may be necessary for restaurant managers to motivate workers toward a culture of adopting positive food safety attitudes. Worker motivation has been associated with desirable outcomes such as having positive food safety attitudes and use of recommended food handling practices (Seaman, 2010). From a public health perspective, regular health inspections of food workers and closure of food premises that do not comply the relevant health and safety standards has been recommended to minimize spread of food‐borne diseases (Woh et al., 2016).

### 3.4 Association between self‐reported and observed food hygiene practices

Table 4 shows the association between self‐reported and observed food hygiene practices. Data from field observations indicate that contrary to self‐reported information, a substantial proportion of food handlers did not use a detergent or disinfectant to wash their hands before food handling or post‐handling potentially contaminated materials (*p* < .05; Table 4). Nonsanitization of hands can contribute to food contamination with pathogenic bacteria (Abdul‐Mutalib et al., 2012). Handwashing with an antibacterial soap can remove over 95% of coliform counts (Toshima et al., 2001). Therefore, improved hand hygiene should be promoted to reduce the risk of transmission of bacterial food‐borne diseases. On the other hand, our findings are consistent with previous food safety studies that showed that food handlers’ self‐reports with respect to the use of desirable hand hygiene practices were largely not supported by findings from observation checklists (Dharod et al., 2007; Reboucas et al., 2016). Self‐reporting has been described as a cognitive measurement and as prone to egocentrism, cognitive bias, and other empowering factors (da Cunha et al., 2019). This highlights the value of observation when studying food hygiene practices of food handlers. Observed practices have been reported to be closer to the actual practice and to be influenced directly by knowledge and indirectly by attitude (da Cunha et al., 2019).

**Table 4:** Association between self‐reported and observed food hygiene practices

Practice	Self‐reported		Observed		χ^2^	*p*
	Yes *n* (%)	No *n* (%)	Yes *n* (%)	No *n* (%)		
Food preparation areas (FPAs) cleaned with at least a detergent prior to food preparation	96 (95)	5 (5)	94 (95)	7 (6.9)	1.39	.240
FPAs rinsed with clean water to remove residues of detergents and disinfectants	94 (93.1)	7 (6.9)	87 (86.1)	14 (13.9)	1.36	.240
Handwashing done using a detergent or disinfectant before handling food	98 (97)	3 (3)	82 (81.2)	19 (18.8)	4.64	.030*
Hands dried using approved method (e.g., disposable towels, air drier, frisk drying)	76 (75.2)	25 (24.8)	43 (42.6)	58 (57.4)	16.24	.001*
Smoking, sneezing and nose‐poking not done when preparing food	77 (76.2)	24 (23.8)	69 (68.3)	32 (31.7)	52.33	.001*
Eating not done when preparing food	77 (76.2)	24 (23.8)	69 (68.3)	32 (31.7)	52.33	.001*
Separate storage of raw and cooked foods	92 (91.1)	9 (8.9)	81 (80.2)	20 (19.8)	29.70	.001*
Separate cold storage of raw vegetables and meat	80 (79.2)	21 (20.8)	71 (70.3)	30 (29.7)	46.90	.001*
Separate cutlery used for raw and cooked foods	60 (59.4)	41 (40.6)	48 (47.5)	53 (52.5)	62.51	.001*
Expired food should not be consumed	99 (98.0)	2 (2.0)	93 (92.1)	8 (7.9)	0.18	.680
Handwashing not done in sinks for washing cutlery	66 (65.3)	35 (34.7)	52 (51.5)	49 (48.5)	50.71	.001*
Cutlery (e.g., knifes, spoons, cups) not handled by surfaces that come into contact with food	97 (96.0)	4 (4.0)	84 (83.2)	17 (16.8)	20.58	.001*
Nonrefreezing of defrosted foods	84 (83.2)	17 (16.8)	77 (76.2)	24 (23.8)	46.90	.001*
Adequate thawing of food	89 (88.1)	12 (11.9)	81 (80.2)	20 (19.8)	34.61	.001*
No hand jewelry (e.g., necklaces, rings and watch)	86 (85.1)	15 (14.9)	77 (76.2)	24 (23.8)	38.48	.001*
Valid food handlers medical exam certificates	93 (92.1)	8 (7.9)	78 (77.2)	23 (22.8)	20.70	.001*
Hand nails kept clean and short	99 (98.0)	2 (2.0)	89 (88.1)	12 (11.9)	2.83	.092
Hair covered with a cap or hairnet	88 (87.1)	13 (12.9)	73 (72.3)	28 (27.7)	24.10	.001*
Apron washed and clean	59 (58.4)	42 (41.6)	45 (44.6)	56 (55.4)	35.72	.001*
Protective gear not taken to potentially contaminated areas (e.g., toilet and home )	90 (89.1)	11 (10.9)	87 (86.1)	14 (13.9)	0.235	.628

* *p* < .05.

A significant proportion of the food handlers did not dry their hands using approved methods such as disposable towels, air drier, and frisk drying (*p* < .05; Table 4). Rather dish towels were used, and in some cases hands were not dried at all. Studies have demonstrated the presence of coagulase**‐**positive staphylococci (Stepanović et al., 2005; Soares et al., 2012) and salmonella (Lee et al., 2017) on food handlers’ hands. Such microbes can be transferred to dish towels during hand drying. About half of the food handlers washed their hands in sinks meant for washing cutlery, which is unhygienic and can contaminate utensils. Many food handlers did not use separate cutlery to prepare raw and cooked foods, contrary to their questionnaire responses (*p* < .05; Table 4). Using the same cutlery such as cutting boards and knives to prepare raw and cooked foods can contribute to cross contamination of food (Abdul‐Mutalib et al., 2012). In addition, there were significant mismatches between self‐reported and observed food handling practices such as smoking, eating, sneezing, and nose‐poking when preparing food (*p* < .05; Table 4). This demonstrates that self‐reports may underestimate the magnitude of undesirable food handling practices. In addition, observed deficits in food handling practices highlight a need for risk‐based food safety training to restaurant supervisors, managers and food handlers.

Field observations showed that 19.8% of food handlers did not adequately thaw frozen food. This percentage is significantly higher than that obtained from self‐reports (11.9%) of same food handlers in the questionnaire study (*p* < .05; Table 4). Food was defrosted either under room temperature or using hot water (> 45°C). Such a practice promotes optimum growth of food spoilage bacteria. It is important that restaurant managers and regulatory agencies encourage use of low refrigerator temperature to defrost frozen food. A substantial proportion of the food handlers refroze defrosted food, contrary to their questionnaire responses (*p* < .05; Table 4). In particular, raw meat was defrosted in bulk in order to remove quantities that were required for the concerned cooking session. The remainder was refrozen. The defreezing–refreezing practice was often repeated each day. This practice increases the microbial load in food (Abdul‐Mutalib et al., 2012; Sani & Siow, 2014), consequently promoting food spoilage and poisoning. Food safety training should emphasize the need for food handlers to predetermine, pack, and freeze separately the calculated amounts food required for each cooking session. This may assist in preventing the unsafe practice of thawing extra food not required. In addition, this study recommends that restaurant food safety managers should consider implementing other preventive measures to minimize the risk of food poisoning to consumers. Such measures include implementing a HACCP system, standard operating procedures (SOPs), the ISO 22000:2018 standard, a total quality management system (TQMS), and good hygiene practices (GHPs).

### 3.5 Correlation among food safety knowledge, attitudes, practices, and work experience

Table 5 presents the correlation among food safety knowledge, attitudes, practices, and work experience among food handlers working in restaurants. Significant positive correlations were observed between food safety knowledge (FSK) and attitudes (*r*_s_ = 0.371, *p* < .05), FSK and SRFHPs (*r* = 0.242, *p* < .05), FSK and observed food handling practices (OFHPs) (*r*_s_ = 0.254, *p* < .05), attitudes and SRFPs (*r*_s_ = 0.229, *p* < .05), and attitudes and OFHPs (*r*_s_ = 0.263, *p* < .05). However, the correlations were not strong (*r*_s_ < 0.4). The findings suggest that the predictors of use of safe food handling practices are food handlers’ FSK and attitudes. Also, FSK appears to contribute positive food safety attitudes. Positive correlations about food handlers’ food safety knowledge, attitudes, and practices are also reported in literature (Abdul‐Mutalib et al., 2012; Sani & Siow, 2014; Al‐Shabib, Mosilhey, & Husain., 2016). In Brazil, de Souza, de Azevedo, & Seabra (2018) reported a positive correlation between food safety knowledge and self‐reported food handling practices. Ncube, Kanda, Mpofu, and Nyamugure (2019) proposed a framework in which food handlers’ food safety knowledge and attitudes are considered to be the major determinants of the use safe food handling practices. Evidently, to improve food handlers’ food safety behaviors, food safety managers must target strengthening the food safety knowledge and attitudes of this category of workers.

**Table 5:** Correlation among food safety knowledge, attitudes, practices, and work experience

Variables	Spearman (*r*_s_)	*p*
Knowledge—attitudes	0.337	.001*
Knowledge—self‐reported food handling practices	0.242	.001*
Knowledge—observed food handling practices	0.254	.001*
Attitudes—self‐reported food handling practices	0.229	.001*
Attitudes—observed food handling practices	0.263	.001*
Work experience—knowledge	−0.001	.990
Work experience—attitudes	0.017	.862
Work experience—self‐reported food handling practices	0.062	.541
Work experience—observed food handling practices	−0.176	.078

A nonsignificant correlation was observed between food handling work experience and all other variables (*p* > .05; Table 5). Abdul‐Mutalib et al. (2012) reported nonsignificant correlations among food handling work experience, knowledge, attitudes, and practices of food handlers. Findings from the current study appear to suggest that work experience does influence food handler's food safety knowledge, attitudes, and practices (Table 5). On the contrary, Nee and Sani (2011) reported a significant relationship between food handlers’ food safety knowledge level and their work experience. Food handlers with greater than six years work experience had significantly higher knowledge when compared to those with less than one year.

### 3.6 Strengths and limitations of the study

This study used a piloted and validated questionnaire whose reliability was satisfactory (k = 0.76–0.94). In addition, each questionnaire was completed in the presence of trained research assistants. Methodological triangulation of self‐reported data concerning the food handling practices was carried out using task performance observations. Furthermore, the study was carried out among restaurant food handlers in the lower‐middle‐income country, which helps to fulfill the lack of research in such contexts. On the other hand, the current study is subject to some methodological limitations. First, we did not examine the knowledge, attitudes, and practices of restaurant managers regarding food safety. Since food safety managers may have significantly higher performance level than food handlers (Illes et al., 2018), our findings may not be generalizable to them. Further research may need to assess the food safety KAP of restaurant managers. Second, cross‐sectional designs lack the capacity to definitely demonstrate cause‐effect relationships (Ncube et al., 2017). Third, a relatively small sample of food handlers used which may affect the external validity of this study. Nonetheless, the sample size is comparable to similar past studies (Abdul‐Mutalib et al., 2012; da Cunha et al., 2014; Martins et al., 2012). Lastly, our study is not immune to sampling bias as purposive sampling was used, which is a nonrandom sampling method.

## 4 CONCLUSION

Results showed significant positive correlation among the food safety knowledge, attitudes, and practices of food handlers. This underscores the need to prioritize improvement of the food safety knowledge and attitudes of food handlers, through measures such as the provision of basic and advanced food safety training programmes, in order to enhance the use of safe food handling practices. Such food safety training programmes should focus on correcting the undesirable practices such as poor hand, cutlery, and working surfaces hygiene, habits (for example coughing and sneezing over food), and inadequate thawing and refreezing of food. Zimbabwe's food safety laws should be revised to include a requirement for managers in the food service sector to provide periodic food safety training to food handlers. In light of the substantial discrepancies between self‐reported and observed food handling practices, our findings demonstrate that self‐reports underestimate the magnitude of undesirable food handling practices. This highlights the need for future food safety studies to use observation to validate food handlers’ self‐reports about their food handling practices. Lastly, restaurant managers should commit themselves to building a positive food safety culture among food handlers.

## CONFLICTS OF INTEREST

All authors declare no conflicts of interest in this article.

## ETHICAL STATEMENT

The study conforms to the Declaration of Helsinki, US and does not involve any human or animal testing. The study protocols and procedures were ethically reviewed and approved by Bindura municipality and the authors’ university.
